# Dissemination of the nurse-administered Tobacco Tactics intervention versus usual care in six Trinity community hospitals: study protocol for a comparative effectiveness trial

**DOI:** 10.1186/1745-6215-13-125

**Published:** 2012-08-01

**Authors:** Sonia A Duffy, David L Ronis, Marita G Titler, Frederic C Blow, Neil Jordan, Patricia L Thomas, Gay L Landstrom, Lee A Ewing, Andrea H Waltje

**Affiliations:** 1 School of Nursing, University of Michigan, 400 North Ingalls Building, Ann Arbor, MI 48109-5482, USA; 2 VA Center for Clinical Management Research, 2215 Fuller Road, Ann Arbor, MI 48105, USA; 3 Department of Psychiatry, University of Michigan, Rachel Upjohn Building, 4250 Plymouth Road, Ann Arbor, MI 48109-2700, USA; 4 Feinberg School of Medicine, Department of Psychiatry and Behavioral Sciences, Northwestern University, Suite 904 Abbott Hall, 710 North Lake Shore Drive, Chicago, IL 60611, USA; 5 Center for Management of Complex Chronic Care, Hines VA Hospital, 5000 South Fifth Ave, Hines, IL 60141, USA; 6 Trinity Health, 34605 Twelve Mile Road, Farmington Hills, MI 48331-3221, USA

**Keywords:** Smoking, Cessation, Inpatient

## Abstract

**Background:**

The objectives of this smoking cessation study among hospitalized smokers are to: 1) determine provider and patient receptivity, barriers, and facilitators to implementing the nurse-administered, inpatient Tobacco Tactics intervention versus usual care using face-to-face feedback and surveys; 2) compare the effectiveness of the nurse-administered, inpatient Tobacco Tactics intervention versus usual care across hospitals, units, and patient characteristics using thirty-day point prevalence abstinence at thirty days and six months (primary outcome) post-recruitment; and 3) determine the cost-effectiveness of the nurse-administered, inpatient Tobacco Tactics intervention relative to usual care including cost per quitter, cost per life-year saved, and cost per quality-adjusted life-year saved.

**Methods/Design:**

This effectiveness study will be a quasi-experimental design of six Michigan community hospitals of which three will get the nurse-administered Tobacco Tactics intervention and three will provide their usual care. In both the intervention and usual care sites, research assistants will collect data from patients on their smoking habits and related variables while in the hospital and at thirty days and six months post-recruitment. The intervention will be integrated into the experimental sites by a research nurse who will train Master Trainers at each intervention site. The Master Trainers, in turn, will teach the intervention to all staff nurses. Research nurses will also conduct formative evaluation with nurses to identify barriers and facilitators to dissemination.

Descriptive statistics will be used to summarize the results of surveys administered to nurses, nurses’ participation rates, smokers’ receipt of specific cessation services, and satisfaction with services. General estimating equation analyses will be used to determine differences between intervention groups on satisfaction and quit rates, respectively, with adjustment for the clustering of patients within hospital units. Regression analyses will test the moderation of the effects of the interventions by patient characteristics. Cost-effectiveness will be assessed by constructing three ratios including cost per quitter, cost per life-year saved, and cost per quality-adjusted life-year saved.

**Discussion:**

Given that nurses represent the largest group of front-line providers, this intervention, if proven effective, has the potential for having a wide reach and thus decrease smoking, morbidity and mortality among inpatient smokers.

**Trial registration:**

Dissemination of Tobacco Tactics for Hospitalized Smokers NCT01309217

## Background

Despite strong evidence for the efficacy of inpatient smoking cessation interventions [1], implementation of smoking cessation in hospitals has been limited. Nurses are ideally positioned to provide smoking cessation interventions to inpatient smokers because: 1) nurses are trained in patient education, psychosocial, and physiological interventions; 2) physician time is at a premium while nursing time is more cost-effective; 3) nurses have both access to patients and the opportunity to develop rapport with them as well as the connection to the physicians within the provider team; 4) nurses understand the patient’s medical condition and can tailor the intervention accordingly; and 5) nurses can read charts, initiate medication orders, and write nursing notes. While a meta-analysis showed nurse-administered cessation interventions are efficacious [2], nurse-administered cessation interventions are seldom implemented due to lack of training and time [3].

Over the last 12 years, our team has developed, tested, and refined the efficacious, nurse-administered Tobacco Tactics intervention in multiple populations including head and neck cancer patients [4], veterans [5], and Operating Engineers (ongoing research). In a recent Veterans Affairs (VA) Service Directed Project [5], our team packaged the Tobacco Tactics intervention into a toolkit and trained 573 inpatient nurses and ancillary personnel in two VA hospitals. Preliminary results show that after adjusting for covariates, there was significant improvement in self-reported six-month quit rates for the pre- versus post-intervention time periods in the intervention as compared to the control site. While we are excited about the rapid dissemination and effectiveness of the Tobacco Tactics intervention in the VA, further rigorous testing of implementation outside the VA system is needed. Using six of forty-seven hospitals in the Trinity Health System, the objective of this quasi-experimental study is to compare the implementation of the nurse-administered Tobacco Tactics intervention in three hospitals compared to usual care in three similar hospitals. The specific aims are to: 1) determine provider and patient receptivity, barriers, and facilitators to implementing the nurse-administered, inpatient Tobacco Tactics intervention versus usual care using face-to-face feedback and surveys; 2) compare the effectiveness of the nurse-administered, inpatient Tobacco Tactics intervention versus usual care across hospitals, units, and patient characteristics using thirty-day point prevalence abstinence at thirty days and six months (primary outcome) post-recruitment; and 3) determine the cost-effectiveness of the nurse-administered, inpatient Tobacco Tactics intervention relative to usual care including cost per quitter, cost per life-year saved, and cost per quality-adjusted life-year saved.

Social marketing theory has been used to design and refine the nurse-administered Tobacco Tactics intervention. Social marketing is the planning and implementation of programs designed to bring about social change using concepts from commercial marketing including the ‘4 Ps’: 1) create an enticing ‘Product’ (that is, packaging the Tobacco Tactics intervention into a toolkit for ease of dissemination); 2) minimize the ‘Price’ the target audience believes it must pay, such as offering continuing education units (CEUs) for training, making it easy for nurses to implement and easy for patients to access; 3) make the exchange available in ‘Places’ that reach the audience and fit its lifestyles, such as providing training as part of scheduled nurse training at hospitals on all shifts; and 4) ‘Promote’ the exchange opportunity with creativity and through channels and tactics that maximize desired responses, such as placing a cessation video on the hospital television station and providing a cleverly illustrated, tailored, Tobacco Tactics patient manual enhanced with medications and telephone follow-up [6].

## Methods/Design

### Design

Since many randomized controlled trials have already tested the efficacy of inpatient smoking cessation interventions [1], the real challenge is integrating these interventions into standard of care. Hence, this effectiveness study will use a quasi-experimental design in six Michigan community hospitals of which three will get the nurse-administered Tobacco Tactics intervention and the other three will provide usual care in accordance to how the hospital responds to current Joint Commission (JC) hospital accreditation standards. Implementing the intervention at the facility (hospital) level was the most likely design to remain sustainable once the study was completed.

Throughout the entire study, smokers are surveyed at baseline, thirty days and six months post-recruitment. In this way, quit rates at two time points for all patients are determined during intervention (of less interest), and post-intervention in both the Tobacco Tactics and usual care groups. There are two sources of control. The first source of control is pre- versus post-intervention changes within hospitals; this source of control will NOT control for seasonal changes. The second source of control is changes in the Tobacco Tactics sites compared to the usual care sites; this source of control WILL control for seasonal changes and any other historical events that may influence quit rates across sites.

The quasi-experimental design allows for implementation in natural environments minimizing threats to external validity as natural environments do not suffer the same problems of artificiality as compared to a well-controlled laboratory setting [7]. While quasi-experimental designs are more feasible, the lack of randomization at the patient level may pose many challenges in terms of internal validity making it hard to rule out confounding variables [8]. To minimize the effect of confounding variables, the six hospitals have been split into two groups of three that as a group are matched on hospital size and percentage of minority patients. While we could have arbitrarily decided which group of three hospitals gets the intervention and which does not, we wanted to reduce investigator bias and used a computerized coin flip to assign the groups to experimental and control conditions. See Table 1 for the overall study design.

**Table 1:** Overall study design

**Intervention**	**Pre staff-intervention quit rate**	**Staff-intervention implementation**	**Post staff-intervention quit rate**
Tobacco Tactics	O_1,_ O_2_	X_1_	O_1,_ O_2_
Usual Care	O_1,_ O_2_		O_1,_ O_2_

*O* = Observation (Thirty-day, six-month).

*X* = Intervention.

### Setting and sample

The setting will be six of forty-seven hospitals in the Trinity healthcare system, which is the fourth largest Catholic health-care system in the United States with facilities in nine states. All units will be included, including general medical/surgical, intensive care units/critical care units (ICU/CCU), psychiatric mental health, obstetrics and gynecology (OB/GYN), and pediatrics. The rationale for including all units results from our VA experience where we found exclusion of specific units to be unsuccessful, as specialty units initially excluded (for example, psychiatric mental health and ICU/CCU) felt discriminated against and felt they needed the intervention at least as much as the other units. Moreover, JC standards do not exempt specialty units therefore inclusion of all units is consistent with real world standards. That said, we recognize the need for adaptation of the intervention to specific units such as OB/GYN or pediatrics where the medication component may not be applicable, but the behavioral component would still apply. Including as many units as possible will increase the generalizability of study findings.

Inclusion criteria for the study include inpatients that: 1) are at least 18 years of age; 2) have smoked a cigarette within one month prior to hospitalization; 3) have a projected hospital stay of at least 24 hours; and 4) are willing to complete the questionnaires. The study will exclude inpatients that: 1) refuse to participate; 2) are involved in a concurrent trial that includes a smoking cessation intervention; 3) are non-English speaking (the intervention is currently only in English); 4) are unavailable to participate (for example, never in the room when the research assistant attempts to consent); 5) are not cognitively able to participate; or 6) are not physically able to participate (see Table 2).

**Table 2:** Eligibility criteria

**Inclusion criteria**	
1. at least 18 years of age	
2. smoked a cigarette within one month prior to hospitalization	
3. projected hospital stay of at least 24 hours	
4. willing to complete the questionnaires	
**Exclusion criteria**	
1. refuse to participate	
2. involved in a concurrent trial that includes intervention on smoking	
3. non-English speaking (the intervention is currently only in English)	
4. unavailable to participate	
5. not cognitively able to participate	
6. not physically able to participate	

### Power analysis

The six Trinity sites for the study (three intervention and three control) employ 3,553 inpatient nurses that work across fifty-eight units and discharge 98,350 patients per year (see Table 3). Of the 98,350 annual patients served by the Trinity hospitals, we conservatively estimate that 80% (78,680) of the inpatients will be well enough to participate and, of these, 20% (15,736) will smoke. Based on our previous studies, we anticipate that 50% will agree to participate for a potential recruitment pool of 7,868, which will be more than enough to recruit the 2,350 smokers needed for 80% power to detect a significant difference in cessation rate (the primary outcome) assuming a 30% cessation rate in the intervention group versus 20% in the usual care group, with an alpha of .05. This power analysis was based on the patient as the unit of analysis with attention to clustering by hospital unit. Effects of the clustering on the power of the study were based on intraclass correlation coefficients (ICC) from our preliminary study and the number of clusters in the design. Using the hospital unit as the unit of clustering was justified given that the study is quasi-experimental rather than experimental and that any other analysis would be underpowered.

**Table 3:** Description of six hospital sites

**Group 1**	**RN positions**	**Number of units**	**Annual discharges**	**% minorities**	**Group 2**	**RN positions**	**Number of units**	**Annual discharges**	**% minorities**
Hospital 1	659	11	19,800	70%	Hospital 4	502	12	18,250	31%
Hospital 2	326	9	17,000	4%	Hospital 5	536	8	20,500	29%
Hospital 3	765	9	11,400	27%	Hospital 6	765	9	11,400	48%
**Total**	**1,750**	**29**	**48,200**	**17,618/48,200 = 37%**	**Total**	**1,803**	**29**	**50,150**	**17,075/50,150 = 34%**

### Procedures

#### Overview

Baseline pre-intervention smoking cessation rates will be obtained in all hospitals (intervention and usual care control). Once baseline quit rates are achieved in all hospitals, the Tobacco Tactics intervention will become standard of care for all inpatient smokers in the intervention hospitals only. That is to say, smokers who refuse to participate in the research will still be offered the Tobacco Tactics intervention. Smokers who are not interested in quitting smoking will at least be given brief advice to quit as this is part of the standard of care Tobacco Tactics intervention. Those smokers that are interested in quitting will be offered the complete Tobacco Tactics intervention. To determine population quit rates, all smokers, regardless of whether they are interested in quitting smoking or not, are offered participation in the study, that is, completing the surveys. Smokers in the control sites will receive usual care and be enrolled in the study to determine their quit rates. Changes in pre- to post-intervention quit rates in the intervention sites will be compared to changes in quit rates in the usual care control sites during the same time period.

#### Enrollment of patients into the study

**Figure 1:**
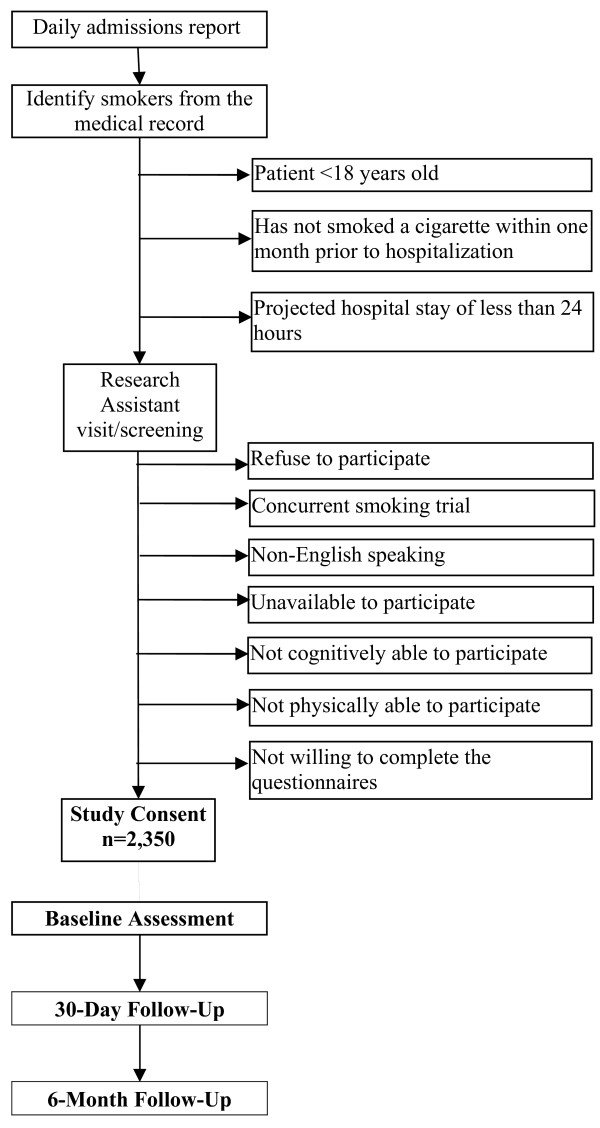
Recruitment flow chart.

Throughout the pre-training, implementation, and post-training period, smokers in all sites will be identified from the nursing assessment at admission and approached by a research assistant to provide written informed consent as was done in our prior studies [9,10]. See Figure 1 for the recruitment flow chart. Participants will be asked to complete a survey about their smoking habits and other potential moderating variables. Research assistants will also complete a medical record data collection form that includes information such as admission and discharge date and diagnoses, comorbidities, length of stay in the hospital, height, weight, and type of insurance.

#### Follow-up

A second shorter survey will be sent to the patient 30 days after being recruited to obtain patient perceptions about cessation services received, satisfaction with services, and smoking status. A longer follow-up survey will be sent six months after recruitment to determine longer-term quit rates. A modified Dillman approach [11] will be used to follow smokers. Long-term follow-up response rates for inpatient smoking studies can be low, ranging from 46% to 62% [12,13]. To minimize missing data, as has been done in our prior studies [5], those who do not respond to the mailed surveys and postcards will be called and offered the opportunity to complete the survey by phone with particular emphasis on the dependent variable (quit versus not quit). Participants will be given $10 for each survey. In addition, to corroborate self-reported smoking status six months post-discharge, all participants will be provided a urinary NicAlert cotinine (a metabolite of nicotine) test strip that can be mailed back with the survey. Those who complete the cotinine test will receive an additional $20 for returning the test. In our prior similar VA study, this was found to be a feasible method for obtaining biochemical verification as 90% of those that returned the six-month survey also returned the urinary cotinine test strip. In this way, quit rates in all sites (Tobacco Tactics and usual care) will be established pre- and post-staff training. See Table 4 for a summary of survey measures.

**Table 4:** General measures and time-points

	**Eligibility criteria log**	**Medical records**	**Baseline**	**thirty-day**	**six-month**
**Independent variable**					
Tobacco Tactics intervention versus Usual Care					
**Control variables**					
**Health behaviors**					
· Nicotine dependence (HSI)*			**X**	**X**	**X**
· Alcohol use (AUDIT-C)*			**X**		**X**
**Clinical characteristics**					
· Comorbidities		**X**			
· Depression (PHQ-2)*			**X**		**X**
**Socio-demographics**					
· Demographics (age, sex, race, educational level, marital status, employment, hospital site)*	**X**	**X**	**X**		
**Dependent variables/outcomes**					
**Aim 1: Provider and patient receptivity, barriers, and facilitators to implementation**					
· Face-to-face feedback	**Ongoing**				
· Surveys	**Pre-, post-, and three months post-training**				
**Aim 2: Cessation efficacy**					
· Thirty-day/six-month* reported smoking				**X**	**X**
· Six-month cotinine test*					**X**
**Aim 3: Cost-effectiveness**					
· Cost per quitter			**X**		**X**
· Cost per life-year saved			**X**		**X**
· Cost per quality-adjusted life-year saved (EQ-5D)			**X**		**X**

* These measures/protocols are common to all CHART studies.

#### Implementation of the Tobacco Tactics intervention

Cochrane Collaboration’s Effective Practice and Organization of Care Group found interventions that are more active, such as educational outreach, train-the-trainer models, and the use of opinion leaders were more effective in changing health-care provider behavior [14]. Consequently, a research nurse will implement the Tobacco Tactics intervention in the experimental sites. The primary role of the research nurse is to work with Master Trainers in each site to implement the intervention. Working with the research nurse, the Master Trainers will prepare the facility for dissemination of the intervention including placing the necessary materials on the units, arranging for easy documentation, and identifying nurse champions (preferably one per unit). One incentive for becoming a unit champion may be an opportunity to participate in a leadership role, which may assist with career promotion. The research nurse, along with the Master Trainers, will provide the training sessions on all shifts until all of the staff nurses are trained. An incentive for staff nurses to attend is the opportunity to obtain one Continuing Education Unit (CEU) needed for re-licensure. Based on our prior experience, it may take from two to six months to train the nurses depending on the degree to which the hospital is able to release them from the unit for training.

#### Identification of barriers and facilitators to implementation

Many barriers and facilitators have already been identified in our VA studies and the Trinity System hospitals will benefit from lessons already learned. However, it cannot be assumed that the intervention can be transferred to the Trinity sites without modification of the implementation strategy, and the types of modifications needed are likely to vary by unit and hospital site. Hence, social marketing will be used to identify barriers and facilitators to implementation of the Tobacco Tactics intervention. This focus on the ‘consumer’ (both the nurses who implement and the patients who receive the intervention) involves in-depth research and constant re-evaluation of every aspect of the program. The staff nurses will be surveyed before training, immediately after training, and again three months after the trainings have been completed to identify barriers and facilitators to implementation which will be kept on a log. The barriers and facilitators will be discussed at research team meetings and modifications to the implementation strategy will be suggested. Moreover, patients will be surveyed thirty days after their release from the hospital to determine their satisfaction with the intervention and whether or not they received specific aspects of the intervention. By utilizing social marketing theory and obtaining feedback from providers, the implementation strategy can be modified to maximize ease of delivery.

#### Sustainability

By participating in process evaluation and actually seeing changes made based on feedback given, we experienced in our VA studies and expect to see in this study, that sustainability will be enhanced because nurses can take ownership of the intervention and are therefore more motivated to keep it going. Master Trainers will ensure that the training is incorporated into the orientation for all new nurses. Ease of nurse documentation will be enhanced by use of a cessation services checklist template.

### Description of the Tobacco Tactics intervention (standard of care when all nurses are trained)

#### Tobacco Tactics toolkit for nurses

For nurses, the cessation toolkit includes: 1) one CEU contact hour for training; 2) a PowerPoint presentation on behavioral and pharmaceutical interventions; 3) a pocket card *Helping Smokers Quit: A Guide for Clinicians* developed by U.S. Department of Health and Human Services, Public Health Service; 4) behavioral and pharmaceutical protocols; and 5) a computerized template for nurse documentation. The PowerPoint presentation covers assessment of smokers, behavioral and pharmaceutical interventions, contra-indications for specific groups of smokers, and case studies. The pocket card provides an overview of behavioral and pharmaceutical protocols, which provide guidance for delivering interventions.

#### Tobacco Tactics toolkit for patients

For patients, the cessation toolkit includes: 1) brochure; 2) videotape; 3) manual; 4) 1-800-QUIT-NOW card; 5) pharmaceuticals; and 6) follow-up phone calls. The brochure is one that we created that includes tips for quitting smoking and additional resources to help patients quit. After reviewing many possible videotapes, we selected *Smoking: Getting Ready to Quit* because it was the most appealing and informative one. The video helps smokers develop the skills they need to quit, as well as provides information about smoking cessation medications and dealing with withdrawal symptoms and potential relapse situations. The Tobacco Tactics manual for patients, which has been tested among head and neck cancer patients and veterans, was highly rated by patients, and has been re-illustrated for a more general population. 1-800-QUIT-NOW is a national portal that connects smokers with the state support quitline. As part of usual care, pharmaceuticals may be provided using an algorithm that uses low-risk options first and progresses to higher-risk options depending on the patient’s smoking history, prior experience with quitting, and contra-indications. Providing the brochure, videotape, and manual in advance of cessation counseling and having a set algorithm for medications will save the nurses’ time at the bedside.

#### Nurse counseling

The Tobacco Tactics intervention begins by having the patient watch the fifteen-minute videotape, which is placed on the overhead television to play at least twice a day, preferably at breakfast and dinner time. The nurse will also provide the patient with the Tobacco Tactics manual to look over. At a later point in time, the nurse will meet with the patient for approximately ten to twenty minutes and provide cessation counseling which can be broken into smaller units (for example, four five-minute sessions) and conducted while providing routine care. The cessation program, based on social cognitive theory [15] and Marlatt and Gordon's relapse prevention model [16], incorporates multiple components deemed necessary for a successful program [17], incorporates Agency for Healthcare Research and Quality (AHRQ) recommendations [18] for treatment of smoking, and tailors the intervention to the individual patient’s medical condition and lifestyle. For example, those with low nicotine dependence may only receive behavioral counseling while those with high nicotine dependence may receive both behavioral counseling and medication. The behavioral component of the intervention can be tailored to the patient and, as outlined in the Tobacco Tactics manual, includes: 1) health consequences of smoking; 2) smoker self-assessment; 3) identifying smoker type; 4) change worksheet; 5) money-saving advantage; 6) goal setting; 7) handling thoughts about smoking; 8) assessing high-risk situations; 9) common triggers; 10) coping with cravings; 11) coping with relapses; 12) benefits of quitting; and 13) guided imagery. The pharmaceutical component of the intervention includes a medication protocol used in our prior studies. Pharmacological management will be initiated by the nurse in consultation with the patient’s physician. This will take place just as any discussions between nurses and physicians occur in the context of the providing of health care to patients in the hospital setting. See Appendix A and Appendix B for an outline of the behavioral and pharmaceutical protocols [5].

#### Physician advice

While this is primarily a nurse-delivered intervention, brief physician advice has been shown to enhance quit rates [19]. In our previous VA study, we considered a number of tactics to encourage physician advice including clinical reminders, which were not acceptable to physicians. Based on this feedback, we have had great success coupling a physician reminder for brief advice along with medication sign-off, which once implemented was very sustainable.

#### Telephone counseling

Studies [20-22] have shown that telephone counseling is efficacious to reinforce the initial intervention visit, promote skills building, and monitor pharmacologic treatment. Yet, our experience has been that, due to cost and procedural barriers, telephone counseling is one of the most difficult components of inpatient cessation interventions to implement and sustain. After exploring several unsuccessful options, we have had great success implementing telephone follow-up through Voluntary Services.

Working with the director of Voluntary Services, we will handpick and train volunteers to provide the telephone cessation counseling. Volunteers will be former (at least one year quit) or non-smokers who have good social skills and are interested in providing direct patient care. Volunteer training consists of: 1) participating in the one-hour Tobacco Tactics training program; 2) viewing the video shown to patients about smoking cessation; and 3) viewing a video *Tools for Being a Helpful Peer Partner* about peer support that has been used in other studies [23]. The video demonstrates three types of communication skills: asking open questions; reflective listening; and encouraging efforts to make change. The video helps the individuals understand that when two people share experiences, it can be a very powerful way for them to help each other cope and make good health decisions. The video describes and models Motivational Interviewing (MI)-style communication skills for peer-to-peer communications, explains the basics of MI-style communications, and provides numerous examples of peer-to-peer conversations that use MI techniques.

Using the Tobacco Tactics manual as a guide, volunteers will be given a script that covers three important aspects of providing support to smokers, namely 1) positive reinforcement; 2) handling thoughts about smoking; and 3) strategies to cope with cravings. Volunteers will be supervised making calls until the Master Trainer is comfortable with their performance and they follow the protocol appropriately. Volunteers will provide only behavioral support and refer all medical questions or unanticipated situations/crises to case managers or 911.

There is a documentation template that allows for the nurse to tick off the components of the intervention provided including counseling and medications. If the nurse ticks off on the documentation template that the patient was given the Tobacco Tactics manual, the medical record is programmed to add the patient’s name and phone number to a list that is forwarded to Voluntary Services on a weekly basis. Volunteers then provide peer telephone cessation counseling to patients at two, seven, fourteen, twenty-one, and thirty days after discharge. Since volunteers cannot access or chart in the medical record, paper documentation can be placed in the paper record or scanned into the electronic record by medical records personnel.

#### Fidelity of intervention

Fidelity checks will be conducted by research staff who will observe 5% of staff and volunteers implementing the intervention. These fidelity checks will establish if the components of the Tobacco Tactics intervention are being implemented. Based on these fidelity checks, changes to the implementation strategy may be introduced.

### Description of usual care

The comparison group will receive usual care [24] in accordance to how the hospital responds to current JC standards. Current JC standards require that inpatient smokers with acute myocardial infarction (AMI), congestive heart failure (CHF), community-acquired pneumonia receive adult smoking cessation advice/counseling. Smoking cessation advice/counseling must include at least one of the following: advice to stop smoking, brochures or handouts on smoking cessation, a smoking cessation aid such as nicotine patch, gum, nasal spray, inhaler, lozenge, or sustained-release bupropion, viewed a smoking cessation video [25]. In the Trinity health-care system, all inpatients are screened for smoking and smokers are given advice to stop smoking.

### Measures

The measures for the CHART study group have been described in detail in the overview article and are summarized in Table 4. In brief, the main outcome measures include self-reported thirty-day point abstinence prevalence at six months and biochemically verified abstinence at six months. Additional measures include the Heavy Smoking Index (HSI) for nicotine dependence [26], the Patient Health Questionnaire 2 (PHQ-2) for depression [27], the Alcohol Use Disorders Identification Test–C (AUDIT-C) for alcohol use [28,29], and demographics (age, sex, race, educational level, marital status, employment, hospital site). At thirty-day follow-up, patients are also asked whether they received specific aspects of the intervention and about their satisfaction with the intervention. The EuroQol (EQ-5D) will be used to calculate cost per quitter, cost per life-year saved, and cost per quality-adjusted life-year saved [30].

Additional process measures, some of which will evaluate sustainability, include facility tobacco performance measures, whether or not materials are being used (for example, Tobacco Tactics manuals), whether the Tobacco Tactics training gets integrated into new nurse training, number of follow-up counseling calls made to patients, nurse confidence in delivering the intervention, nurse self-report that they are continuing to implement at three-month post-training, and patient report of, receipt of, and satisfaction with the components of the intervention. Some sample questions include: ‘How confident are you in your abilities to provide smoking cessation services to smokers?’ ranging from ‘Not at all confident’ to ‘Extremely confident’ and ‘Please indicate why you do not expect to provide these services’ with choices of ‘Lack of confidence,’ ‘Not enough training,’ ‘Not enough time,’ ‘Hesitant to upset patients,’ ‘Not my job,’ and ‘Other’. Post-intervention measures will be compared to pre-intervention measures both within and across sites.

### Data analysis

All analyses that compare the two treatment groups will use the intent-to-treat approach in which patients’ data are analyzed based on the treatment intended, not on the treatment actually received. This approach is taken because it is the best method for evaluating the potential effects of disseminating the Tobacco Tactics intervention, which is our focus. Another implication of this approach is that the approximately 25% of patients who are lost to follow-up are included which assures that differences between conditions are not actually due to differential drop out. Extensive follow-up will be conducted including by telephone to minimize the amount of missing data. Statistically justified imputation methods such as multiple imputation will be used to allow analyses including those who have dropped out.

Analyses will adjust for clustering for patients within units using generalized estimating equations (GEE) analyses. GEE is a method of analysis that can account for the correlations among responses from patients in the same cluster [31]. Given the structure of the study with patients nested within hospital units, the hospital unit will be treated as the cluster. The correlation matrix will be of the exchangeable form, assuming the same correlation among any pair of patients in the same hospital unit. This is standard structuring of the matrix for analyzing clustered non-longitudinal data. Given concerns that GEE might be biased with a small number of units, the adjustment for that factor recommended by Morel [32] will be used for each GEE analysis.

There is concern that individual hospitals might differ in factors that influence implementation and effectiveness of the intervention. Though the study would be underpowered to use the hospital as the unit of clustering, several strategies will be used to counter the effects of hospital differences on our finding. If there are baseline differences in the type of patients among the six hospitals, we can control for these differences by adding these variables as covariates in the analysis. If there are multiple differences and we are underpowered to add all of these variables as covariates, we will create a propensity score to control for these differences. Note the quit rates and other factors will be assessed in all hospitals before the intervention is conducted in any of them. Comparison of the post-intervention quit rates to the pre-intervention quit rates (and post and pre levels of other outcomes) is built into the analyses.

#### Aim 1: Determine provider and patient receptivity, barriers, and facilitators to implementing the nurse-administered, inpatient Tobacco Tactics intervention versus usual care using face-to-face feedback and surveys

Descriptive statistics (means and frequency distributions) will be used to summarize the nurses’ survey results. Patient participation rates will be calculated by dividing the number of inpatient smokers that participated by all smokers screened and found to be eligible to participate. Descriptive statistics will be calculated to determine if smokers were offered specific cessation services, whether they participated in these services, and their satisfaction with these services. Satisfaction rates of 80% are anticipated because our similar in-clinic smoking cessation intervention with head and neck cancer patients had similar rates [4]. To test for differences between the Tobacco Tactics and usual care sites in services received (yes/no) and satisfaction with those services (scale of one to five), GEE analysis using a link function of logit (for dichotomous dependent measures) or identity (for quantitative dependent measures) with adjustment for the clustering of patients within units will be conducted. GEE analysis is designed for data that are collected in clusters (the hospital units) and works with data with a wide variety of distributions.

#### Aim 2: Compare the effectiveness of the nurse-administered, inpatient Tobacco Tactics intervention versus usual care across hospitals, units, and patient characteristics using thirty-day point prevalence abstinence at thirty days and six months (primary outcome) post-recruitment

Patients will report on their smoking status (thirty-day point prevalence) at thirty days and six months (primary outcome variable) post-recruitment. At six months, all study participants will be asked to return the completed cotinine test. Descriptive statistics (percentage quit for seven and/or thirty days at six months) will be computed and reported for the hospitals and units in each condition. The standard errors will be computed taking into account the clustering of patients within units. GEE analyses analogous to logistic regression, but adjusting for the clustering of patients within units, will be used to compare the pre- and post-intervention quit rates in the intervention as compared to the usual care control hospitals. Recall that in every hospital, quit rates will be assessed both before and after implementation of the intervention (whether or not the hospital is among those receiving the intervention). Whether the baseline quit rates differ or not between hospitals, baseline quit rates will be considered in analyses to meet this objective.

Smoking quit rates will be assessed throughout the implementation phase to assess whether cessation rates are improving; however, the primary outcome is the smoking cessation rates at the time when the intervention is independently being conducted by hospital staff. Descriptive statistics will be computed and reported. Preliminary analyses will use chi-square tests of association to compare the quit rates in the hospitals in each condition.

We anticipate a quit rate of 30% in the hospitals receiving the Tobacco Tactics intervention and 20% in the usual care hospitals. However, to assure that these differences are not just due to differences between the hospitals, a GEE analysis will be conducted using the logit link function with the hospital unit as the definition of the cluster analogous to moderated logistic regression analysis in which quitting is predicted by time frame (baseline versus intervention period), intervention group (Tobacco Tactics versus usual care), and the product of those two variables. The statistical significance of the product will indicate that the difference between the Tobacco Tactics and the usual care comparison sites depends on whether or not the intervention has been implemented. We anticipate no difference between the sites at baseline, but a notable difference after the interventions have been implemented.

In addition, harm reduction will be compared between the two groups by analyses of number of cigarettes smoked per day, number of quit attempts, and nicotine dependence (as indicated by Fagerstrom test). For each of these analyses, the same general approach taken to the primary dependent variable will be applied including: descriptive statistics on baseline and intervention period and a GEE with the hospital unit as the definition of the cluster analogous to moderated regression analysis to compare the differences between the sites (Tobacco Tactics versus usual care) by time (baseline versus intervention period). Both the descriptive statistics and the significance tests differ for these other outcome measures because of their different distributions (that is, normal or not). Count variables will be analyzed assuming a Poisson distribution using a log link function while the Fagerstrom test scores will be modeled with an identity link function since it is normally distributed.

Other analyses related to aim 2 will test the moderation of the effects of the interventions by selected patient characteristics. We hypothesize that patients will be more likely to quit if they have higher confidence in ability to quit, higher education level, lower addiction, lower alcohol intake, and a smoking-related diagnosis such as heart disease. We will also test whether the effects of the specific intervention (Tobacco Tactics or usual care) received are moderated by these factors. All of these effects will all be tested by GEE analyses analogous to moderated regression analyses (whether logistic, linear, or Poisson) including product terms to test the interaction of these factors with the intervention received.

#### Aim 3: Determine the cost-effectiveness of the nurse-administered, inpatient Tobacco Tactics intervention versus usual care including the cost per quitter, cost per life-year saved, and cost per quality-adjusted life-year saved

Using both societal and health system perspectives, cost-effectiveness will be assessed by constructing three ratios. The incremental cost-effectiveness ratio (ICER) is the difference in average costs between the Tobacco Tactics and the usual care groups divided by the difference in average effectiveness between the two groups. First, we will calculate the cost per quitter (incremental cost of achieving cessation for those in the Tobacco Tactics intervention compared to usual care). The numerator will be calculated by subtracting the cost of usual care from the cost of the Tobacco Tactics intervention, and the denominator will be based on the difference in quit rates between the two groups. No discount rate will be applied since both the costs and quits will occur within the same period of time. Second, we will estimate the cost per life-year saved, defined for this study as the cost per lifetime quitter divided by the number of life-years saved per lifetime quitter. The potential number of life-years saved will be obtained using published data and the use of Monte Carlo simulation. A similar procedure will also be used to estimate the cost per quality-adjusted life-year (QALY) [33]. A discount rate of 3% will be applied to the estimated life-years and quality-adjusted life years saved since current life-years are generally considered to be of greater value than future life-years [34]. Sensitivity analyses will be used to test other input values in the life-years and QALYs models (for example, 1% or 5% rather than 3% discount rate, other estimates of relapse rates). Both short-term costs (during the intervention period) and long-term costs (downstream total medical care costs, including pharmacy, inpatient, and outpatient costs) will be calculated.

## Discussion

The nurse-administered Tobacco Tactics intervention is innovative in that, aside from having all of the expected components of guideline recommendations [18] for behavioral and pharmaceutical cessation interventions, social marketing strategies have been used to obtain consumer feedback to develop the intervention and materials, which patients and staff have found to be engaging. The Tobacco Tactics intervention has been packaged into a toolkit for nurses and patients, which makes it easy to implement and export to other facilities. By training all nurses, the largest group of front-line providers, there is a possibility for a wide reach of the intervention.

The study design is novel in that it implements the intervention at the facility level, which enhances sustainability when the study ends and minimizes cross-contamination between intervention and comparison arms. Comparing the Tobacco Tactics intervention to ‘real world’ usual care will provide for careful testing of the intervention. The sample is novel in that it uses a network of community hospitals that may serve as an avenue for broader dissemination at the end of the study. Implementation strategies, many recommended by providers themselves, make the Tobacco Tactics intervention easy to integrate into busy inpatient units.

The design is in concert with a recent Institute of Medicine (IOM) report which discusses the need for direct comparison of effective interventions in typical day-to-day clinical care including smoking cessation interventions [35]. Moreover, the design is consistent with the recommendations in the American Recovery and Reinvestment Act of 2009 which suggests studying outcomes of the organization and delivery of care including translation and dissemination efforts ensuring that evidence from comparative effectiveness research is accessible to and usable by patients and other consumers, providers, payers, and policy makers [36]. Priorities cited by this act include increasing the capacity for practical experimental and quasi-experimental comparative effectiveness studies; emphasizing the evaluation of broad, health system-level strategies to improve the quality and value of care; identifying subgroups of patients most likely to benefit from a given intervention; and increasing the emphasis on developing and evaluating strategies to disseminate research results and encourage the use of evidence in the care of individuals and patient populations [37]. By assigning hospitals rather than patients to conditions, we are really testing the applicability of the intervention in real world settings.

The idea behind implementing the Tobacco Tactics program at the hospital level is that, as we move toward population-based public health approaches to address smoking, our goal is to get the largest number of quits given a fixed unit of investment. The return on investment is a function of the potential reach of the program, the effectiveness of the program, and the cost of delivering the program. What we are looking for is high reach, high effectiveness, and low cost. Imagine the enormous reach and public health impact of an inpatient smoking cessation intervention if all of the largest group of front-line providers, namely nurses, were trained to effectively provide the Tobacco Tactics intervention.

## Trial status

Human studies approval was obtained from all participating sites. As of May 2012, 651 participants out of 2,350 have been recruited. Recruitment is ongoing.

## Appendix A. Smoking cessation behavioral management protocol

1. Assess if patient interested in quitting.

2. If patient not interested, leave brochure at bedside.

3. If patient interested, leave brochure and arrange for patient to view videotape.

4. After videotape, provide patient with patient manual to read if able.

5. Using patient manual, assist patient with behavioral intervention including:

a. self-assessment,

b. smoker type,

c. smoking costs,

d. handling cravings,

e. relapse prevention, and

f. medication options.

6. Along with patient, identify and arrange for cessation medications (see pharmaceutical protocol).

7. Arrange for follow-up calls [5].

## Appendix B. Smoking cessation pharmaceutical management protocol

1. Recommend nicotine replacement (patch, gum, or lozenge) if:

a. never used patch, gum, or lozenge before,

b. used patch, gum, or lozenge successfully in the past (smoke-free >3 months).

2. Recommend nicotine replacement (patch AND gum OR lozenge) if:

a. smoke greater than one pack per day, and

b. failed nicotine replacement therapy in past.

3. Recommend Bupropion if:

a. failed nicotine replacement therapy in the past (smoke-free <3 months)

b. patch, gum, or lozenge intolerant (that is, rash, and so on), and

c. history of depression or currently has depressive symptoms.

4. Recommend combination nicotine replacement (patch, gum, or lozenge) and bupropion if:

a. failed nicotine replacement and bupropion monotherapy in the past.

5. Recommend varenicline if:

a. intolerance or treatment failure to nicotine replacement and bupropion [5].

## Competing interests

The authors declare that they have no competing interests.

## Authors’ contributions

All authors agreed on the need for a protocol paper. SD conceived of the study and drafted the paper. DR was responsible for the data analysis section. LE and AW were responsible for working out many of the details for implementation. MT was responsible for establishing access to the Practice-Based Research Network. FB assisted with strategies for implementation. NJ is responsible for the cost-effectiveness analysis. PT and GL are our practice partners and have assisted with implementation strategies. All authors reviewed the manuscript and provided extensive feedback. All authors read and approved the final manuscript.
